# PI3Kγ Drives Priming and Survival of Autoreactive CD4^+^ T Cells during Experimental Autoimmune Encephalomyelitis

**DOI:** 10.1371/journal.pone.0045095

**Published:** 2012-09-13

**Authors:** Iain Comerford, Wendel Litchfield, Ervin Kara, Shaun R. McColl

**Affiliations:** The Chemokine Biology Laboratory, the School of Molecular & Biomedical Science, the University of Adelaide, Adelaide, South Australia, Australia; University of Muenster, Germany

## Abstract

The class IB phosphoinositide 3-kinase gamma enzyme complex (PI3Kγ) functions in multiple signaling pathways involved in leukocyte activation and migration, making it an attractive target in complex human inflammatory diseases including MS. Here, using *pik3cg* ^−/−^ mice and a selective PI3Kγ inhibitor, we show that PI3Kγ promotes development of experimental autoimmune encephalomyelitis (EAE). In *pik3cg^−/−^* mice, EAE is markedly suppressed and fewer leukocytes including CD4^+^ and CD8^+^ T cells, granulocytes and mononuclear phagocytes infiltrate the CNS. CD4^+^ T cell priming in secondary lymphoid organs is reduced in *pik3cg^−/−^* mice following immunisation. This is attributable to defects in DC migration concomitant with a failure of full T cell activation following TCR ligation in the absence of p110γ. Together, this results in suppressed autoreactive T cell responses in *pik3cg^−/−^* mice, with more CD4^+^ T cells undergoing apoptosis and fewer cytokine-producing Th1 and Th17 cells in lymphoid organs and the CNS. When administered from onset of EAE, the orally active PI3Kγ inhibitor AS605240 caused inhibition and reversal of clinical disease, and demyelination and cellular pathology in the CNS was reduced. These results strongly suggest that inhibitors of PI3Kγ may be useful therapeutics for MS.

## Introduction

Multiple Sclerosis (MS) is the most common inflammatory disorder of the central nervous system (CNS) and is a chronic, debilitating and demyelinating disease. Following breakdown of immunological tolerance to CNS antigens by unknown mechanism(s), T and B cells invade the CNS initiating the accumulation of innate immune effector cells within the brain and spinal cord. The resulting inflammation leads to CNS demyelination, oligodendrocyte loss, axonal degeneration and impaired nervous system function [1]. Autoimmune responses similar to those in MS are commonly modelled using experimental autoimmune encephalomyelitis (EAE) in mice, which allows experimental dissection of the molecular mechanisms driving CNS autoimmunity [2]. In active EAE, autoreactive encephalitogenic Th1 and Th17 CD4^+^ T cells are activated through immunisation, invade the CNS and subsequently promote the recruitment of immune effector cells such as monocytes and neutrophils, resulting in demyelinating autoimmune inflammation that resembles human MS in many clinical and histopathological features. This model is commonly used to assess the importance of molecular and cellular components in CNS autoimmunity and provide proof-of-concept for novel therapeutics for human MS [2], [3]. For example, sphingosine-1 phosphate receptor agonists and antibody-mediated blockade of the α4-integrin were shown to be effective inhibitors of EAE [4], [5], subsequently leading to the development of Fingolimod and Natiluzimab respectively, as current MS therapeutics used in the clinic. However, while these therapies are proving of some benefit in treating MS, more effective therapies are required which necessitates identification and evaluation of novel therapeutic targets.

Phosphoinositide-3-kinases (PI3Ks) are a large family of dual-specificity lipid and protein kinases known mainly for their role in catalysing the phosphorylation of phosphatidylinositol-4,5-biphosphate (PIP_2_) to phosphatidylinositol-3,4,5-triphosphate (PIP_3_), a key second messenger that recruits PH-domain containing proteins, such as Akt, to the plasma membrane to initiate signal transduction cascades important for cell proliferation, migration and survival [6]. Class IA PI3Ks are generally activated downstream of receptor tyrosine kinases (RTKs) whilst the class IB PI3K (PI3Kγ) is activated by G-Protein coupled receptor (GPCR) triggering. Our previous work identified a role for the class IA PI3K, PI3Kδ, in promoting Th17 responses during EAE [7]. Unlike the two other class I isoforms of PI3K that are essential for life (PI3Kα and PI3Kβ) [8], [9], PI3Kδ and PI3Kγ are not essential for development and display a restricted pattern of expression, mainly confined to cells of the immune system where they contribute to the control of leukocyte activation and migration [10], [11], [12], [13]. Consisting of a catalytic p110γ subunit and two regulatory subunits (p101 and p84), PI3Kγ has been shown to play an important role in promoting migration and activation of various leukocyte subsets including neutrophils, mononuclear phagocytes and some lymphocytes following activation downstream of GPCRs, such as chemokine receptors [13], [14], [15], [16]. Thus, PI3Kγ has been shown to make an important contribution to the pathogenesis of several animal models of human inflammatory diseases including rheumatoid arthritis (RA), asthma, systemic lupus erythematosus (SLE) and diabetes [17], [18], [19], [20], [21], [22] and is under investigation as a drug target for a variety of human inflammatory disorders [23]. Therefore, it is likely that PI3Kγ inhibition has the potential to attenuate MS. Here we have used *pik3cg^−/−^* mice, which lack expression of p110γ, and a highly selective PI3Kγ inhibitor to conduct a detailed investigation of the role of this protein in CNS autoimmune disease and we show that PI3Kγ plays a critical role in EAE by controlling CD4^+^ T cell activation and survival.

## Results

### Lack of p110γ Prevents Development of EAE

**Figure 1:**
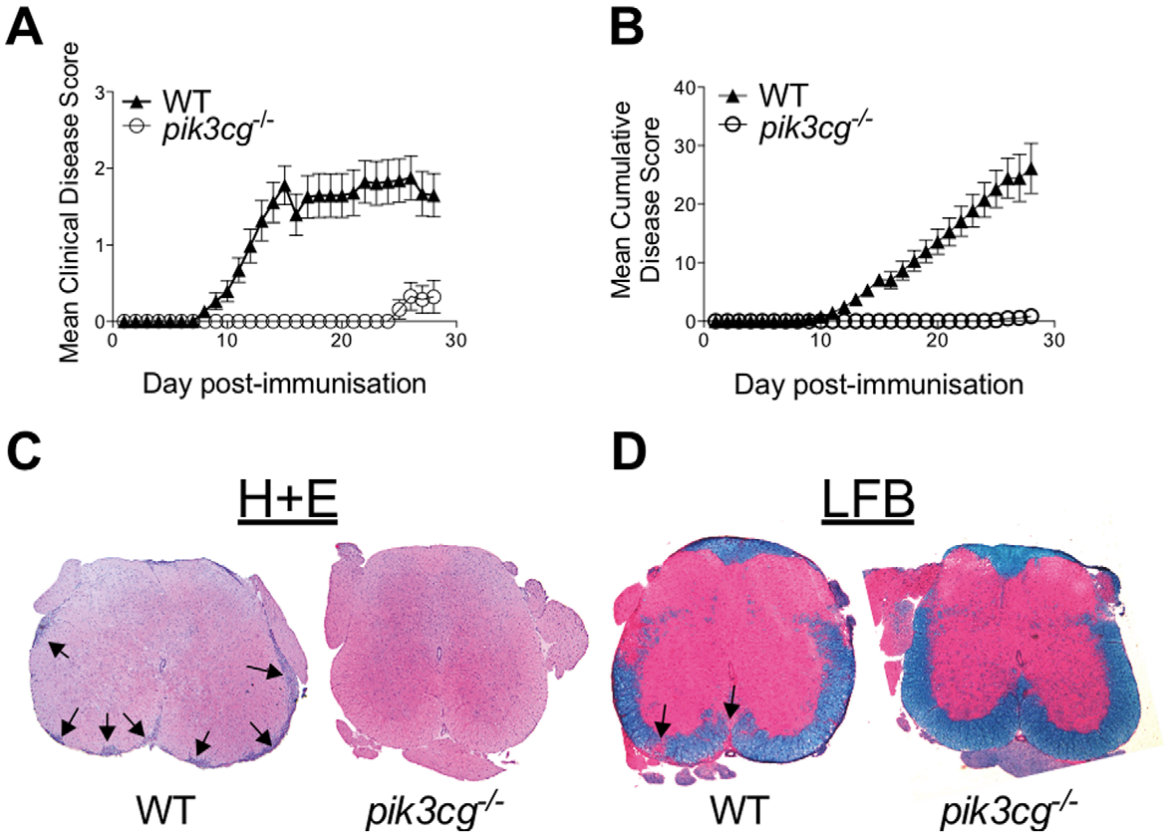
EAE is suppressed in *pik3cg^−/−^* mice. (**A**) EAE was induced in *pik3cg^−/−^* (n = 21) and WT (n = 19) mice. Clinical disease scores are plotted as mean ± s.e.m. from each mouse. Data is pooled from 3 independent experiments. (**B**) Clinical disease scores expressed as cumulative disease scores over time. (**C**) Histology of spinal cords from *pik3cg^−/−^* and WT mice at day 15 post-immunization for EAE. As indicated, sections were stained with haematoxylin and eosin (H+E), luxol fast blue, haematoxylin and eosin (LFB/H+E). Images are representative of those taken from multiple sections. Arrows indicate lesions or demyelinated areas.

To investigate the role of PI3Kγ in CNS autoimmune disease, EAE was induced in WT and *pik3cg^−/−^* mice, which lack the catalytic subunit of PI3Kγ, p110γ. A profound inhibition of clinical disease was apparent in *pik3cg^−/−^* mice (Figure 1A and B). Remarkably, while the incidence of disease in WT mice was 100%, greater than 75% of the *pik3cg^−/−^* mice immunized for EAE remained completely free of clinical disease signs within the 4 weeks following disease induction and, in those that developed EAE, clinical signs were mild and onset was substantially delayed compared with WT mice (Table 1). Histological analyses of spinal cord tissue taken on day 15 post-immunisation when peak disease was apparent in WT mice revealed that *pik3cg^−/−^* mice lacked the characteristic lesions containing infiltrating leukocytes, while these were clearly apparent in WT spinal cord sections (Figure 1C). Furthermore, luxol fast blue staining of spinal cord from mice immunized for EAE revealed no evidence of CNS demyelination in *pik3cg^−/−^* mice whilst this was an obvious feature in WT spinal cord sections (Figure 1D). These data clearly indicate that p110γ plays a key role in driving demyelinating autoimmunity in the CNS.

**Table 1:** Effect of deletion of p110γ on EAE.

Genotype	Incidence	Lethality	Day of Onset	Maximum Disease Score	Mean Cumulative Disease Score
WT	100% (19/19)	0% (0/19)	11.5 (±0.55)	2.33 (±0.24)	26.1 (±14.8)
pik3cg^−/−^	23.8% (5/21)***	0% (0/21)	26.4 (±0.05)***	0.31 (±0.17)***	0.85 (±2.0)***

Data are shown as mean ± s.d. Day of disease onset is the day an animal first displayed clinical signs of EAE. Maximum disease score is the highest clinical disease score recorded. Mean cumulative disease score is the accumulated total disease score obtained by mice taken to day 28 post-immunisation. Data is pooled from 3 independent experiments.

### Reduced Immune Priming of CD4^+^ T Cells during EAE in the Absence of p110γ

**Figure 2:**
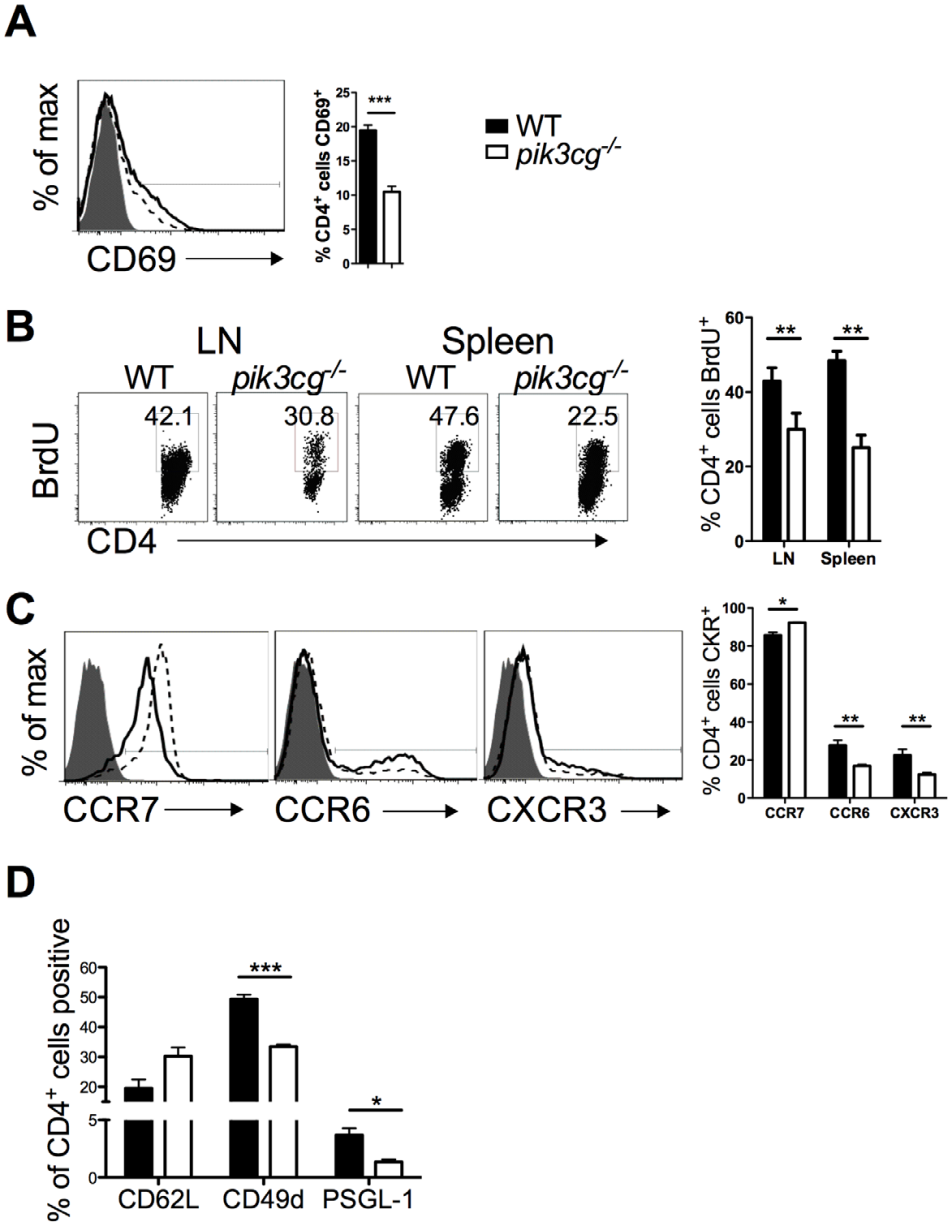
Priming of *pik3cg^−/−^* CD4^+^ T cells is reduced during EAE. (**A**) Frequencies of CD4^+^ cells in draining LN that are CD69^+^ on day 9 post-immunisation for EAE as determined by flow cytometry. A representative histogram overlay gating on CD4^+^ cells is shown (filled = isotype control on WT, solid line = anti-CD69 on WT, dotted line = anti-CD69 on *pik3cg^−/−^*) (**B**) *In vivo* proliferation of CD4^+^ cells, measured by BrdU incorporation, is reduced in *pik3cg* ^−/−^ mice at day 9 post-immunization for EAE. (n = 6 mice per group). Representative dot plots gating on CD4^+^ cells are shown. (**C**) Expression of chemokine receptors by CD4^+^ T cells indicative of T cell activation was determined by flow cytometry. Representative histogram overlays showing expression of CCR7, CCR6 and CXCR3 are shown (filled = isotype control, solid line = WT, dotted line = *pik3cg^−/−^*). (**D**) Expression of CD62L, CD49d and PSGL-1 by CD4+ cells from spleen of day 9 immunised mice (n = 3 mice per group). All data shown are mean ± s.e.m. (*, p<0.05).

**Figure 3:**
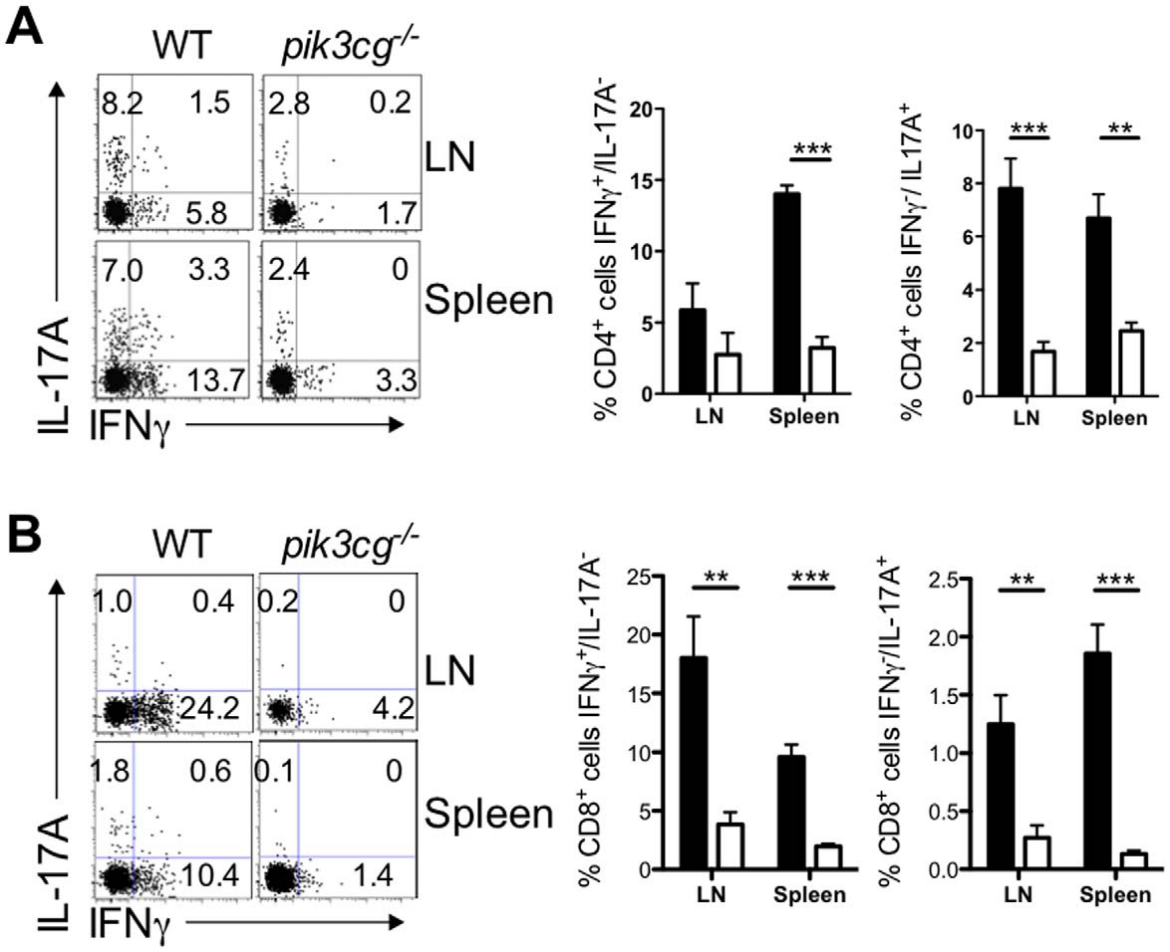
Reduced priming of cytokine-producing CD4 and CD8 T cells during EAE in ***pik3cg^−/−^*** **mice.** (**A**) The proportion of Th1 (IFN-γ^+^/IL-17^−/^CD4^+^ cells) and Th17 (Th17 (IFN-γ^−/^IL-17^+^/CD4^+^ cells) cells in the draining LNs and spleen was assessed by isolating lymphocytes at days 9 and 15 post-immunization for EAE and staining for surface CD4 and intracellular cytokines. Representative flow cytometric dot plots of LN and spleen CD4^+^ cells are shown. (**B**) The proportion of Tc1 (IFN-γ^+^/IL-17^−/^CD8^+^ cells) and Tc17 (IFN-γ^−/^IL-17^+^/CD8^+^ cells) cells in the draining LNs and spleen was assessed by isolating lymphocytes at days 9 and 15 post-immunization for EAE and staining for surface CD8 and intracellular cytokines. Representative flow cytometric dot plots of LN and spleen CD8^+^ cells are shown. All data shown are mean ± s.e.m. n = 6 mice per group. (*, p<0.05).

EAE is a CD4^+^ T cell-driven autoimmune disease [24]. Therefore the nature and magnitude of CD4^+^ T cell responses occurring in secondary lymphoid organs of *pik3cg^−/−^* and WT mice was compared. While no differences in CD4^+^ T cell frequencies in secondary lymphoid organs of WT or *pik3cg^−/−^* mice at resting state were detected (data not shown), clear defects in T cell priming following EAE immunization were apparent in *pik3cg^−/−^* mice. Surface expression of the early activation marker CD69 on CD4^+^ T cells following immunization was reduced by approximately 50% in the absence of p110γ (Figure 2A) and this corresponded with a notable reduction in CD4^+^ T cell proliferation in both LN and spleen following immunization in *pik3cg^−/−^* relative to WT mice (Figure 2B). Expression of additional CD4^+^ T cell activation markers CD44 and CD86 was also reduced in *pik3cg* ^−/−^ mice compared with WT (data not shown). Furthermore, chemokine receptors expression by CD4^+^ T cells was also examined following EAE immunization and *pik3cg* ^−/−^ mice displayed higher frequencies of CD4^+^ T cells positive for the homeostatic chemokine receptor CCR7 than WT mice and lower frequencies positive for the inflammatory chemokine receptors CCR6 and CXCR3 (Figure 2C). In addition, expression of the adhesion molecules CD62L, PSGL-1 and VLA-4 on CD4^+^ T cells also differed between WT and *pik3cg* ^−/−^ mice following immunization for EAE. Specifically, we measured a trend for the LN-homing selectin CD62L to be present on a higher proportion of CD4^+^ T cells from *pik3cg* ^−/−^ mice than WT (although this did not reach statistical significance), while the selectin PSGL-1 and integrin CD49b (VLA-4) were expressed on lower proportions of CD4^+^ T cells in *pik3cg* ^−/−^ mice than WT (Figure 2D), indicative of reduced CD4^+^ T cell activation in these mice. Furthermore, we also measured markedly reduced frequencies of memory (CD44^hi^/CD45RO^+^) CD4^+^ T cells in *pik3cg* ^−/−^ mice relative to WT 28 days after immunization for EAE (data not shown). Clear deficiencies in cytokine production by *pik3cg* ^−/−^ CD4^+^ T cells were also apparent. Frequencies of IFNγ-producing Th1 and IL-17A-producing Th17 cells in both LN and spleen were reduced by 2 to 5-fold in *pik3cg* ^−/−^ mice compared with WT following immunization for EAE (Figure 3A). Also notable was an almost complete lack of highly pathogenic CD4^+^ T cells that express both IFNγ and IL-17A in *pik3cg* ^−/−^ lymphoid organs, whilst these were present in WT (Figure 3A). A marked reduction in Tc1 and Tc17 cells was also measured in the CD8^+^ T cell compartment of *pik3cg* ^−/−^ mice compared with WT following immunization for EAE (Figure 3B). Collectively, these data demonstrate that T cell priming during EAE is profoundly diminished in the absence of PI3Kγ signals and that *pik3cg* ^−/−^ CD4^+^ T cells do not acquire the surface phenotype, trafficking capability or the proliferative capacity of encephalitogenic WT CD4^+^ T cells and do not produce cytokines that contribute to EAE pathogenesis.

### p110γ is Required for Optimal Dendritic Cell Migration and T Cell Activation

**Figure 4:**
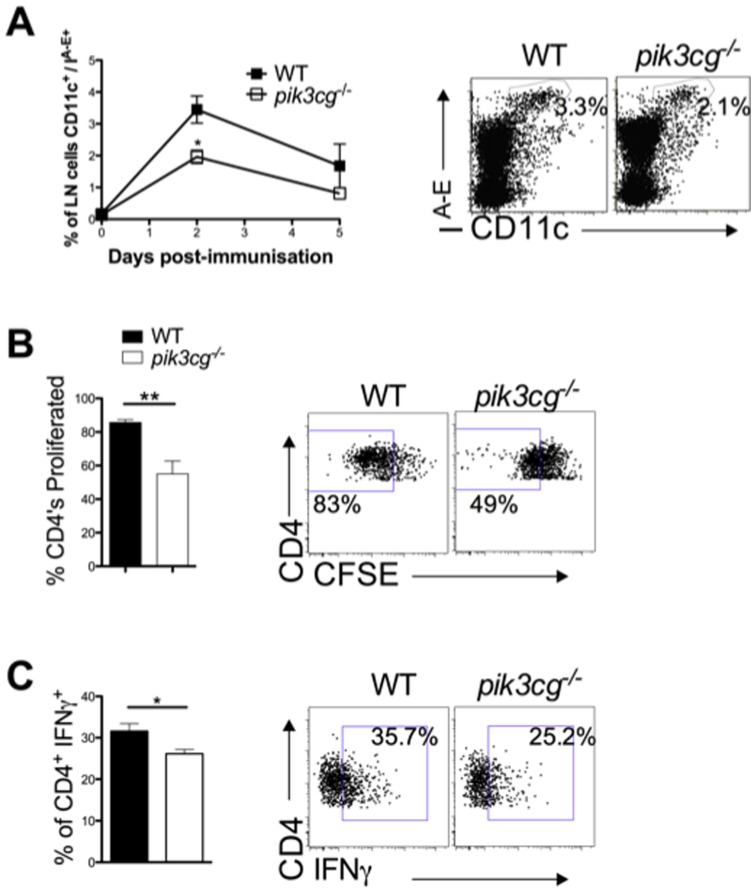
Dendritic cell migration and *in vitro* T cell activation are reduced in the absence of PI3Kγ signaling. (**A**) DLNs from WT and *pik3cg^−/−^* mice were harvested either prior to, or 2 or 5 days-post immunisation for EAE, collagenase treated and CD11c^+^/MHCclassII^hi^ cells enumerated by flow cytometry. Representative dot plots gating on migratory DCs are shown. (**B**) Proliferation of *pik3cg^−/−^* CD4^+^ cells *in vitro* is inhibited following TCR stimulation. Splenocytes were harvested from naïve WT and *pik3cg^−/−^* mice, labelled with CFSE and cultured on anti-CD3 coated tissue culture trays for 72 hours in the presence of anti-CD28 antibodies. Cells were then harvested from culture and divided CD4^+^ cells enumerated by flow cytometry. Data shown is from 6 mice per group. (**C**) Production of IFNγ by *pik3cg^−/−^* CD4^+^ cells is reduced following *in vitro* TCR stimulation. Splenocytes were harvested from naïve WT and *pik3cg^−/−^* mice and cultured on anti-CD3 coated tissue culture trays for 72 hours in the presence of anti-CD28 antibodies. Cells were then harvested from culture and intracellular IFNγ detected in live CD4^+^ cells following 4 hours activation with PMA/ionomycin in the presence of golgistop and enumerated by flow cytometry. Data shown is from 6 mice per group. All data shown are mean ± s.e.m. (*, p<0.05).

The evident defects in CD4^+^ T cell priming apparent *pik3cg^−/−^* secondary lymphoid tissues may be explained by two, not necessarily mutually exclusive, mechanisms: a failure of antigen-presenting cell (APC) function and/or an intrinsic defect in T cell activation. A role for PI3Kγ has been implicated in both of these processes by other investigators [13], [14], [25]. Therefore, these possibilities were examined in the context of the EAE model. Time-course analysis of DC trafficking to the draining LN (DLN) following subcutaneous immunisation for EAE revealed that in *pik3cg* ^−/−^ mice, CD11c^+^/MHC class II^hi^ DCs were less abundant than in WT mice 48 hours following immunization (Figure 4A), although normal frequencies of DCs have been reported to reside in the skin of these mice [14]. This suggests that p110γ is required for optimal DC migration to secondary lymphoid organs during EAE. Next, we tested CD4^+^ T cell functionality in these mice. *In vitro* activation of T cells via anti-CD3 crosslinking revealed that *pik3cg* ^−/−^ CD4^+^ T cells had a reduced capacity to proliferate (Figure 4B) and produce IFNγ than WT CD4^+^ T cells (Figure 4C), indicating an intrinsic role for p110γ in TCR signaling. Together these data indicate that PI3Kγ signaling is required for triggering of the immune system through at least two separate mechanisms: by promoting efficient DC trafficking and by promoting T cell activation, in terms of proliferation and cytokine production.

### CNS Infiltration of Pathogenic Leukocyte Populations is Reduced and CD4+ T Cell Apoptosis Increased in pik3cg^−/−^ Mice

**Figure 5:**
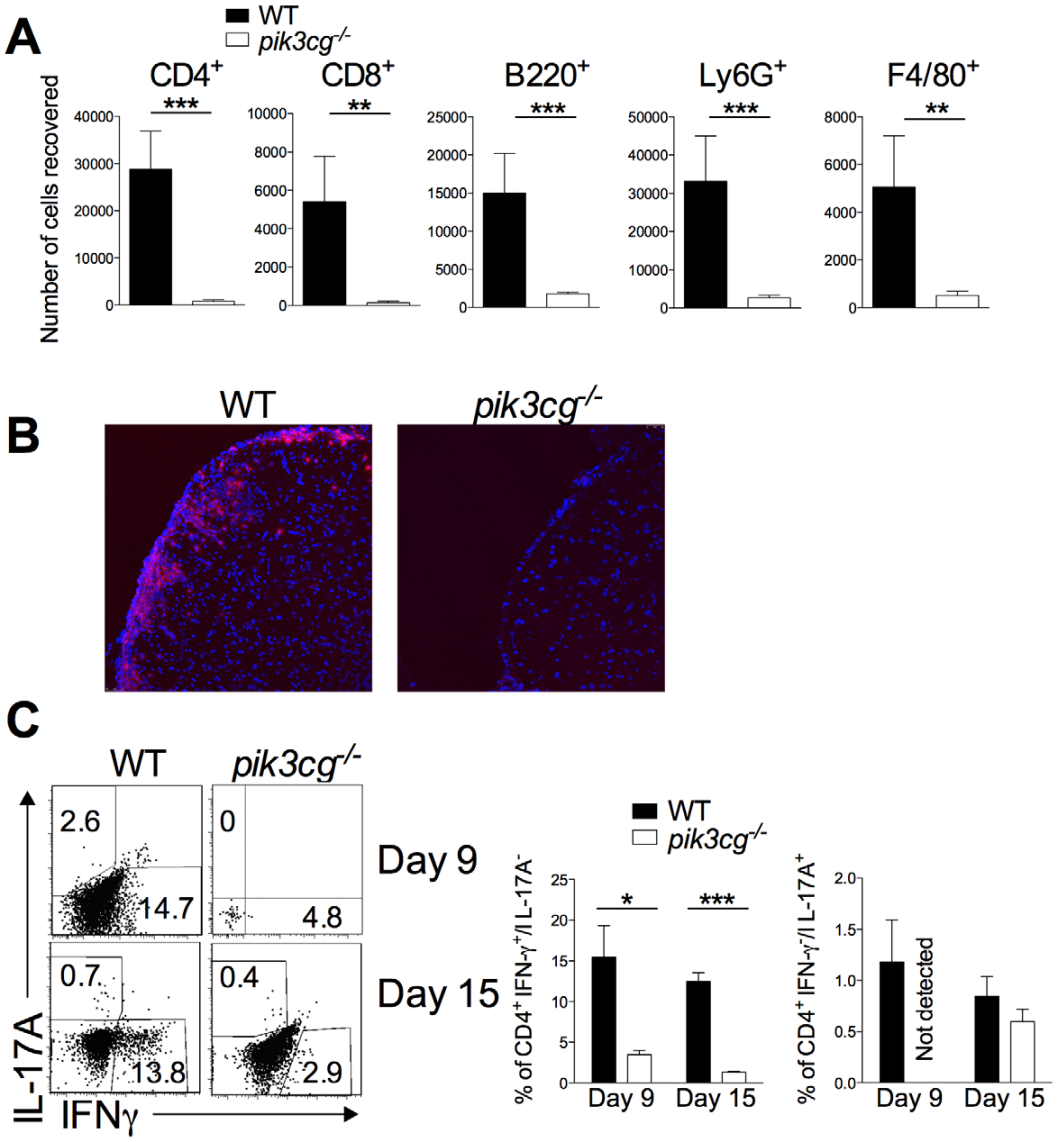
Reduced leukocyte infiltration and Th1 and Th17 responses in the CNS of *pik3cg^−/−^* mice during EAE. (**A**) The number of CD4^+^, CD8^+^, B220^+^, Ly6G^+^ and F4/80^+^ cells in the spinal cord on day 15 post-immunisation was enumerated by flow cytometry (n = 6 mice per group). (**B**) Sections of spinal cord from *pik3cg^−/−^* and WT mice at day 15 post-immunization for EAE were prepared and immunofluorescence for CD4 performed (CD4 = red, DAPI = blue). Images are representative of those taken from multiple sections. (**C**) The proportion of Th1 (IFN-γ^+^/IL-17^−/^CD4^+^ cells) and Th17 (Th17 (IFN-γ^−/^IL-17^+^/CD4^+^ cells) cells in the spinal cord was assessed by isolating lymphocytes at days 9 and 15 post-immunization for EAE and staining for surface CD4 and intracellular cytokines. Representative flow cytometric dot plots of spinal cord CD4^+^ cells are shown. All data shown are mean ± s.e.m. n = 6 mice per group. (*, p<0.05).

**Figure 6:**
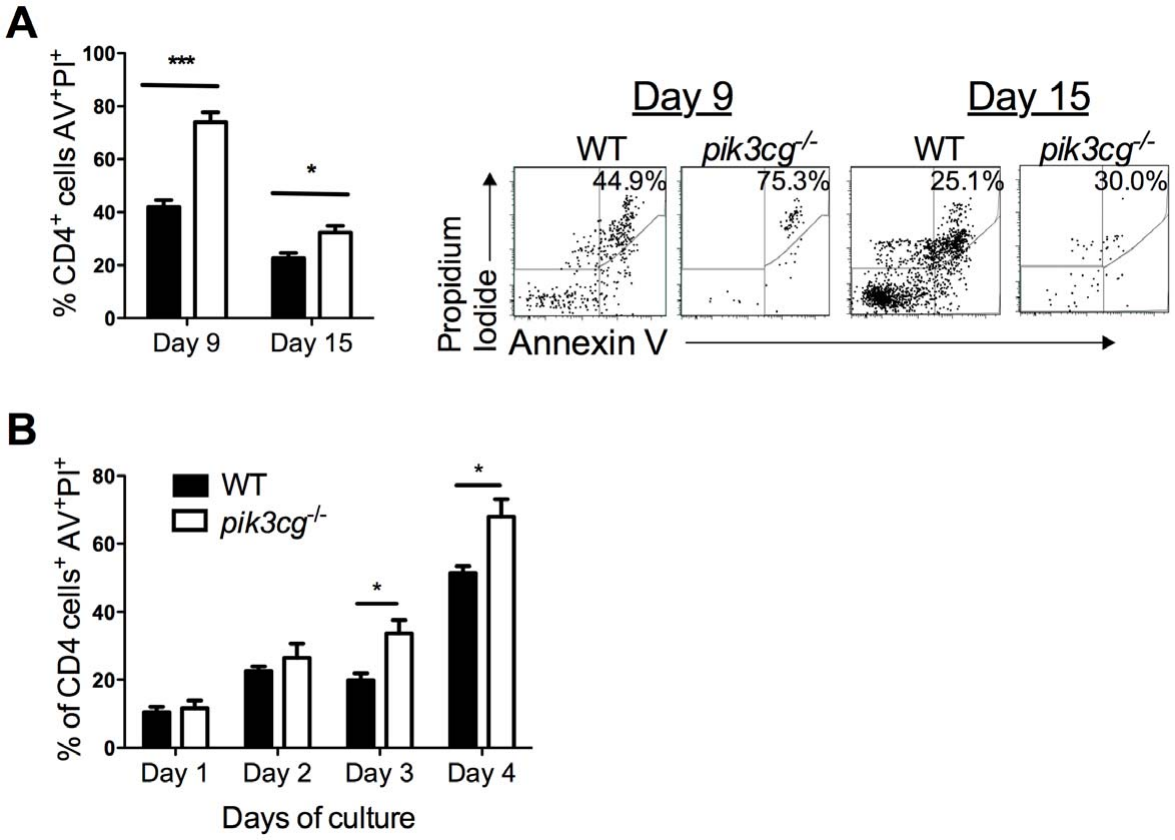
Increased CD4^+^ T cell apoptosis in *pik3cg^−/−^* **mice during EAE.** (**A**) The proportion of apoptotic (Annexin V^+^/Propidium iodide^+^) cells in the spinal cord was higher in *pik3cg^−/−^* mice compared with WT. Representative dot plots gating on CD4^+^ lymphocytes harvested from spinal cord taken from mice on day 9 and day 15 of EAE are shown. (**B**) Increased activation induced cell death in *pik3cg^−/−^* CD4^+^ T cells. Splenocytes were harvested from naïve WT and *pik3cg^−/−^* mice and cultured on anti-CD3 coated tissue culture trays for 4 days in the presence of anti-CD28 antibodies. Every 24 hours from day 1 onward, cells were collected and analysed for the presence of apoptotic (Annexin V^+^/Propidium iodide^+^) CD4^+^ T cells by flow cytometry. In all cases 5–8 mice were included in each group. Data are mean ± s.e.m.

Next, the consequence of these defects on infiltration of the CNS by immune effector cells following EAE induction in WT and *pik3cg^−/−^* mice were investigated. Flow cytometric analysis revealed profoundly reduced frequencies of CD4^+^ and CD8^+^ T cells, B cells, macrophages and neutrophils in the spinal cord of *pik3cg^−/−^* mice compared with WT (Figure 5A). Furthermore, CD4^+^ cells could clearly be detected within perivascular cuffs and the parenchyma of WT spinal cord, but were not detectable in *pik3cg^−/−^* mice (Figure 5B). Th17 and Th1 cells that invade the CNS have a major role in initiating and maintaining disease pathogenesis [26]. When frequencies of these cells in the CNS was examined after EAE induction, clear reductions in Th1 cells were apparent in *pik3cg^−/−^* mice at both day 9 and day 15 post-immunisation. Th17 cells were not detectable on day 9 post-immunisation in *pik3cg^−/−^* spinal cords but were present at equivalent frequencies on day 15 post-immunisation (Figure 5C). We hypothesized that these evident reductions in cytokine-producing CD4^+^ T cells present in the CNS of EAE-immunised *pik3cg^−/−^* mice were, at least in part, caused by increased T cell apoptosis resulting from the lack of PI3Kγ signaling. To address this, apoptotic CD4^+^ T cells in the CNS of mice immunized for EAE on day 9 and day 15 were enumerated by flow cytometry. At both time-points, significantly increased proportions of CD4^+^ T cells were apoptotic in spinal cords of *pik3cg* ^−/−^ compared to WT mice (Figure 6A). This increase in apoptosis was specific to the CD4^+^ compartment as CD45R^+^ and CD8^+^ cells had equivalent frequencies of apoptotic cells between *pik3cg* ^−/−^ and WT (data not shown). Furthermore, results from *in vitro* polyclonal T cell activation following CD3 cross-linking indicated that CD4^+^ T cells from *pik3cg* ^−/−^ mice died more rapidly of apoptosis than those from WT mice (Figure 6B). Together, these studies using GM mice lacking PI3Kγ signaling strongly suggest that this enzyme plays a critical role in CNS autoimmunity by promoting autoreactive T cell responses.

### Oral Administration of a PI3Kγ Inhibitor Prevents Development of EAE

**Figure 7:**
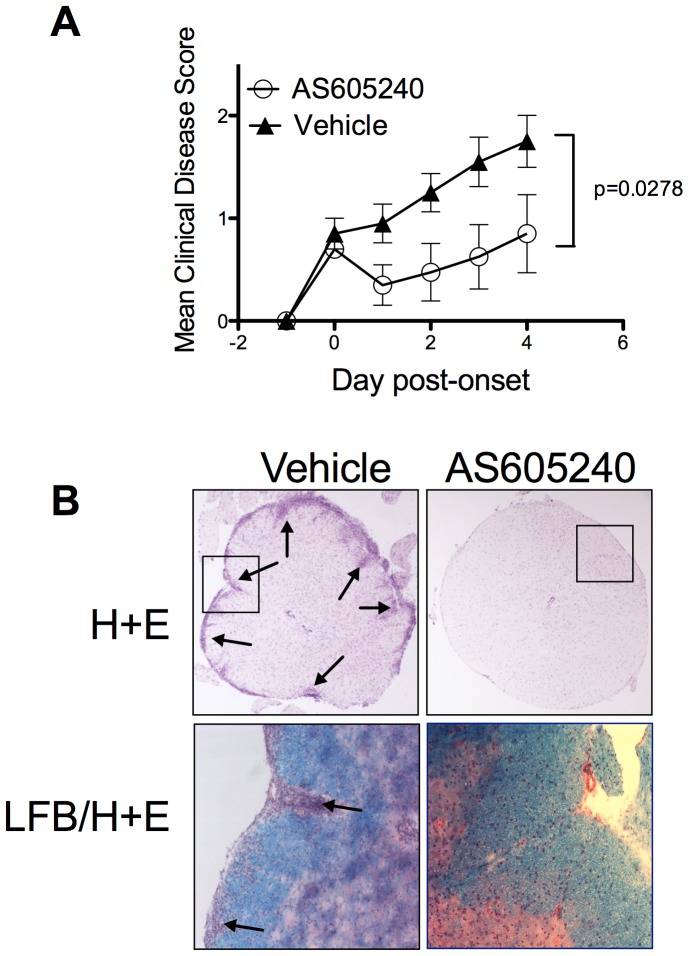
Treatment with an orally active PI3Kγ inhibitor inhibits EAE pathogenesis. EAE was induced in WT mice and these were treated with 30 mg/kg AS605240 or vehicle control by oral gavage beginning on the day of disease onset on a mouse-to-mouse basis. (**A**) Clinical disease scores are plotted as mean ± s.e.m. from each mouse from the day of disease onset. Data is pooled from 2 independent experiments using a total of 10 mice per group. (**B**) Histology of spinal cords taken from AS605240 treated and vehicle only treated mice at the end of the treatment period. As indicated, sections were stained with haematoxylin and eosin (H+E), luxol fast blue, haematoxylin and eosin (LFB/H+E). Images are representative of those taken from multiple sections from 5 mice per group. Arrows indicate lesions or demyelinated areas.

To address whether PI3Kγ is a good candidate drug target for CNS autoimmunity, the efficacy of an orally active PI3Kγ inhibitor at treating EAE was assessed. Mice were immunized for EAE and on the day of clinical disease onset were treated with 30 mg/kg of AS605240 (a highly selective PI3Kγ inhibitor) [18], [19], or vehicle control, by oral gavage. Remarkably, 60% of the AS605240-treated mice fully recovered from EAE and displayed no more clinical signs during the course of the treatment (Table 2). All vehicle-only treated mice continued to succumb to clinical EAE. Significantly reduced clinical disease scores and cumulative disease scores were apparent in AS605240-treated mice (Figure 7A and Table 2). Histological analysis of the CNS of these mice at the end of the treatment period revealed that the AS605240-treated group had reduced cellular pathology in the spinal cord and reduced signs of demyelination compared with the control group (Figure 7B).

**Table 2:** Effect of AS605240 treatment on EAE.

Treatment	% Recovery	Mean Cumulative Score	Mean Peak Score
Vehicle	0% (0/10)	6.35 (±0.85)	1.95±0.77
AS605240	60% (6/10)*	3.0 (±1.11)*	0.9±1.17*

Mice were treated with 30 mg/kg AS605240 by oral gavage beginning on the day of disease onset on a mouse-to-mouse basis. 2 independent experiments, using a total of 10 mice per group, were performed. Recovery indicates the percentage of mice that returned to a clinical score of 0 after disease onset. Peak disease represents the first day an animal reached its highest recorded clinical disease score and data are shown as mean ± s.d.

## Discussion

In this manuscript we demonstrate that PI3Kγ signalling plays a major role in the development of EAE and provide evidence supporting the notion that PI3Kγ inhibitors may be useful therapeutics in demyelinating human autoimmune diseases such as MS. Mice lacking PI3Kγ signalling capacity are markedly protected from the clinical signs of EAE and have reduced histopathological signs of demyelinating CNS autoimmunity. This is likely due, at least in part, to the combined effect of multiple defects that were measured in the activation of the immune system when PI3Kγ signalling is absent, including reduced DC migration, reduced TCR-induced activation of CD4^+^ T cells and enhanced CD4^+^ T cell apoptosis. The efficacy of an orally active PI3Kγ inhibitor in a therapeutic setting was also tested, revealing that PI3Kγ inhibition prevents further development of EAE after the onset of clinical symptoms and reverses clinical disease.

The mechanism of inhibition of EAE when PI3Kγ is deleted or inhibited is likely to be due to combined disruptions to leukocyte migration, activation and survival. A role for PI3Kγ in DC trafficking from skin to the DLN has been previously reported [14] and our results are consistent with this defect in *pik3cg^−/−^* mice influencing the outcome of EAE immunisation. As peripheral presentation of CNS auto-antigen is also a feature of MS [27], this may have implications for targeting PI3Kγ in MS. However, this DC migratory defect was only partial and is unlikely to completely account for the substantial decrease in clinical EAE measured here in *pik3cg^−/−^* mice. The possibility that additional defects in leukocyte migration, particularly the migration of antigen-specific T cells to the CNS, contribute to the effect of deleting or inhibiting PI3Kγ during EAE is likely, however this was not fully addressed in the present study because following immunisation, *pik3cg^−/−^* CD4^+^ T cells expressed reduced levels of inflammatory chemokine receptors, and were inadequately primed, making execution and interpretation of adoptive transfer EAE experiments using T cells from *pik3cg^−/−^* mice problematic. The reduced CXCR3 measured on CD4^+^ T cells during EAE in *pik3cg^−/−^* mice is in line with a recent study by Barbi et al., who demonstrated that induction of CXCR3 expression on T cells is PI3Kγ-dependent [28], although the reduced priming of CD4^+^ T cells measured in the present study may also contribute to our observation. Our data therefore also suggest that expression of CCR6 on CD4^+^ T cells may have a PI3Kγ-dependent component although this was not tested directly in the present study. Nevertheless, a defect in leukocyte migration contributing to the reduced pathology of EAE in *pik3cg^−/−^* mice is highly likely and supported by our observations of reduced *in vitro* migration of *pik3cg^−/−^* compared with WT lymphocytes to both homeostatic (CCL19 and CXCL12) and inflammatory (CXCL11 and CCL20) chemokines (data not shown). We, and others, have shown that the CCR6/CCL20 axis is involved in the pathogenesis of EAE [29], [30], [31], and that blockade of CXCR3 inhibits entry of encephalitogenic CD4^+^ T cells into the CNS [32], [33]. PI3Kγ has been shown to be essential for chemokine-driven migration and adhesion of neutrophils [10], [13], [16], [34], monocytes/macrophages [10], [15], [35] and eosinophils [36], although its essential contribution to lymphocyte migration appears to be highly cell type- and chemoattractant receptor-specific. While B cell migration appears to be largely PI3Kγ independent, with PI3Kδ the major contributor [37], T cell migration has been shown to both PI3Kγ-dependent [37], [38] and –independent [39], [40], [41] by various investigators. Taken together with the results of the present study, it seems likely that migration into the CNS of encephalitogenic T cells and other effector leukocytes is reduced during EAE as a result of PI3Kγ deletion or inhibition. Indeed, we also measured reduced CD8^+^ T cell activation as a result of PI3Kγ deletion and, whilst this may have limited significance during EAE, these cells have been strongly implicated in the pathogenesis of MS.

The defects in *pik3cg^−/−^* T cell activation measured in the present study are likely due to the defects in DC migration described above, coupled with defective T cell proliferation and activation following TCR triggering, a process in which conflicting results have been published with respect to the contribution of PI3Kγ. Perhaps surprisingly, given that TCR signal transduction leads directly to activation of RTK signalling and PI3Kγ is activated primarily via GPCR stimulation, a role for PI3Kγ in T cell activation both *in vitro* and *in vivo* has been clearly demonstrated previously [13], [25]. Following TCR activation, PI3Kγ has been reported to be activated following association with Ga_q/11_, ZAP-70 and lck, although whether these interactions are direct or indirect remains to be determined [25]. However, conflicting data suggesting that TCR signalling is PI3Kγ independent also exist. Normal T cell activation was reported in *pik3cg^−/−^* mice during adjuvant-induced arthritis [42] and equivalent naïve CD4^+^ T cell activation was reported between WT and *pik3cg^−/−^* T cells induced both *in vitro* and *in vivo* using TCR-transgenic mice [38] and in a related study by Berod et al. [43]. It seems likely that these apparent discrepancies are due to differences in the T cell activation systems and different read-outs of activation that have been utilised between these different studies. Irrespective of this, GPCR ligands have been reported to influence T cell proliferation, co-stimulation, cytokine production and survival [44], [45], [46], [47] and it is unlikely that GPCR activation is necessarily equivalent from one T cell activation system to another, which tend to vary in the type of TCR-induced stimulation, costimulatory signals provided and the source and purity of naïve T cells used. Resulting variations in GPCR expression or ligand abundance could conceivably influence the nature of crosstalk between different immuno-receptor signalling systems engaged in both PI3Kγ-dependent and -independent signals during T cell activation, leading to the diverse outcomes reported regarding the contribution of PI3Kγ in TCR signalling and PI3Kγ-mediated signals may differentially influence proliferation, cytokine production, survival and memory development of T cells. Importantly, we have measured all of these functional readouts of T cell activation in the present study, which lends weight to our conclusion that PI3Kγ does indeed play an important role in T cell activation. In addition, crosstalk and direct physical interactions between RTKs and GPCRs [48] and in particular the TCR and GPCRs such as CXCR4 have been reported [49] and it is likely that PI3Kγ makes a substantial contribution to signalling downstream of these complexes. Therefore, the diversity in expression of chemokine receptors and other GPCRs amongst different T cell subsets may also contribute to the apparently variable contribution made by PI3Kγ in T cell activation, although this remains to be determined in detail.

This study expands on two recent reports that investigated the role of PI3Kγ in CNS inflammation [43], [50]. Consistent with our results, both reported reduced CNS pathology during experimental encephalomyelitis in mice lacking p110γ. Rodrigues et al., reported reduced EAE in *pik3cg* ^−/−^ mice associated with reduced expression of the monocyte-attracting chemokines CCL2 and CCL5 in the CNS, a finding that fits well with our observation that recruitment of inflammatory cells to the CNS is markedly reduced in *pik3cg* ^−/−^ mice. Furthermore, Rodrigues et al., also reported an increase in apoptotic cells in the CNS of *pik3cg* ^−/−^ mice using a TUNEL-based histological approach. Here, we extend their findings by demonstrating that it is CD4^+^ T cells that die more readily by apoptosis at this site, a finding in keeping with a previous report implicating PI3Kγ in memory T cell survival [51] and similar to our findings using PI3Kδ-deficient mice [7]. Berod et al., also reported reduced EAE in *pik3cg* ^−/−^ mice that was associated with reduced T cell priming in secondary lymphoid tissues. Furthermore, they also provided evidence that pharmacological inhibition of PI3Kγ with AS605420 reduced EAE, although the level of disease inhibition reported in that study was substantially less than that observed in the present study in which 60% of the animals exhibited complete recovery. This is most likely due to the alternative routes of administration of the AS605240 inhibitor employed. In the present study, oral administration of AS606240 was used rather than intraperitoneal injection as performed by Berod et al. Because it has previously been determined that the oral route of administration leads to a prolonged *in vivo* half-life of this inhibitor when compared with intravenous delivery [18], it is likely that sustained levels of AS605240 are required for effective treatment of EAE which is better achieved by oral administration of AS605240 than by the intraperitoneal route.

At present there are six disease-modifying therapies approved for use in MS patients (interferon-alpha, interferon-beta, mitoxantrone, glatiramer acetate, natalizumab and fingolimod) [52], [53]. While useful, these therapies are of limited efficacy and can cause significant adverse side effects. Moreover, none are effective in treating progressive MS. Therefore more effective and complementary MS therapies are required. The results of the present study along with various biochemical and pharmacological studies indicate that PI3Kγ may be a good drug target in MS, due to its involvement in numerous distinct biochemical processes relevant to this disease, including regulating leukocyte migration, activation and survival [10], [13], [51], [54]. It is likely however that PI3Kγ only controls a proportion of each of those processes. A highly selective PI3Kγ inhibitor would therefore be expected to dampen, rather than completely inhibit, these events. The advantage of targeting PI3Kγ may therefore reside in the fact that mild simultaneous inhibition of several processes involved in the pathogenesis of a complex disease such as MS may effectively reduce disease without significant adverse side effects. Therefore, while PI3Kγ has been shown to make an important contribution in several models of human inflammatory disease including RA [17], [18], SLE [19], diabetes [20], asthma [21], [22] and now MS, whether or not this enzyme will make a safe and effective drug target in these human diseases should now be determined.

Previously we demonstrated that the class IA PI3Kδ also plays an important role in EAE [7]. It remains to be determined whether PI3Kγ and PI3Kδ play redundant or distinct roles in the pathogenesis of EAE. However, it is likely that these enzymes play distinct roles given the clearly divergent modes of activation of these enzymes (GPCR- vs tyrosine kinase-mediated respectively) and the disparate immune/inflammatory signalling pathways in which these enzymes have been implicated (e.g. PI3Kγ: neutrophil migration, neutrophil respiratory burst, mast cell degranulation, DC migration and T cell activation; PI3Kδ: B cell migration, B cell activation, CD28 signalling, Th17 differentiation) [7], [23]. Mice lacking both PI3Kγ and PI3Kδ fail to develop peripheral T cells due to impaired thymocyte development [55], [56], [57], making analysis of EAE in mice double deficient in these enzymes highly problematic. Recently, a new class of PI3K inhibitors that block both PI3Kγ and PI3Kδ have been developed [58], [59] and follow up studies will determine whether these are even more potent than PI3Kγ inhibitors alone in treating EAE. Therefore the results of this study, along with our previous demonstration that PI3Kδ also plays an important role in EAE, suggest that dual PI3K inhibitors may be particularly effective in MS.

## Materials and Methods

### Animals and Pharmacological Inhibitors

*pik3cg^−/−^* mice on a C57Bl6 background [10] were obtained from Professor Shaun Jackson, Monash University, Melbourne, and bred at the University of Adelaide Animal House. Age and sex-matched C57BL/6 mice were purchased from The University of Adelaide Animal House, South Australia or the Australian Research Council and housed at the University of Adelaide Animal House. AS605240 was purchased from Sigma.

### Ethics Statement

All experimental protocols used in this study were approved by the University of Adelaide Animal Ethics Committee. At all times the principals of reduce, refine and replace were adhered to and animal suffering was kept to a minimum.

### Induction of EAE

Female wild-type (WT) C57BL/6 or *pik3cg^−/−^* were immunized subcutaneously in the hind flanks and at the scruff of the neck with a total of 25 µg Myelin Oligodendrocyte Glycoprotein (MOG) peptide-_35–55_ (GL Biochem, China) emulsified in 120 µl Complete Freund’s Adjuvant (CFA) as previously described [60]. CFA was prepared by mixing 15% mannide manooleate (Sigma), 85% mineral oil (Sigma) and 8.33 mg/ml *M. tuberculosis* (Difco Laboratories) and grinding extensively using a mortar and pestle. Two hours prior to immunization, and two days after, the mice were intravenously injected with 300 ng of pertussis toxin (List Biological Laboratories Inc., California, USA) in endotoxin-free PBS. Clinical EAE was evaluated on a scale of 0–5: 0, normal; 0.5, slight tail weakness; 1, tail weakness 2, hind limb weakness or a flaccid tail, 2.5 tail paralysis and abnormal gait; 3, hind limb paralysis; 4, hind and partial fore limb paralysis; 5, moribund.

### Collection of Tissues

Mice were euthanized, the right atrium cut and then perfused through the left ventricle with PBS as previously described [7], [60], before the draining LNs (inguinal and brachial) and the spinal cord removed. Tissue samples were embedded in Tissue-Tek® OCT embedding medium (Sakra Finetek), frozen and sectioned. In some instances, cell suspensions were prepared from the draining LNs, spleens and spinal cords for flow cytometric analysis. Cells were prepared as previously described [7], [60]. For analyses of DCs in LNs, LNs were minced with a scalpel and then treated with Type II Collagenase for 30 minutes at 37°C prior to cell suspension preparation

### Immunolabeling Cells for Flow Cytometry

Flow cytometric labeling of cell surface antigens was performed essentially as previously described [7], [60]. In experiments where intracellular cytokines were detected, cells were activated for 4 hours at 37°C in RPMI containing 20 ng/ml PMA (Sigma), 1 µM ionomycin (Invitrogen) and Golgistop™ (BD Biosciences). Prior to addition of antibodies, Fc-receptors were blocked by incubating cells in PBS/1% BSA/0.04% sodium azide (PBA) containing 200 µg/ml mouse-gamma globulin (Rockland). Following staining of cell surface antigens, cells were fixed and permeabilized using a Cytofix/Cytoperm kit (BD Biosciences) according to the manufacturer’s instructions. The following antibodies were used in this study (all from BD Biosciences): anti-CD4:PE (553652), anti-CD4:Alexa Fluor 647 (557681), anti-CD4:PE-Cy7 (552775), anti-CD11c-PE (553802) and anti-IFN-γ-FITC (554411) and anti-IL-17A-PE (559502), anti-I^A-E^:biotin (553622), anti-CD69:PE-Cy7 (552879), anti-CD8:Alexa Fluor 647 (557682) anti-CD45R:PE-Cy5 (553091), anti-Ly6G:FITC (551460), anti-CD49d:PE (557420), anti-CD62L:PE (553151) and anti-CD62P:FITC (550866). Anti-mCXCR3 (MAB1685) and anti-mCCR6 (MAB590) antibodies were purchased from R&D Systems. Anti-F4/80:FITC (11-4801) was purchased from eBiosciences. Anti-mCCR7 (clone 4B12) was purified from hybridoma supernatant. Unconjugated primary antibodies were detected by using an anti-RatIgG:Alexa 647 secondary antibody (Molecular Probes A21247). In instances where unconjugated primary antibodies were detected using anti-rat secondary antibodies, an additional blocking step in 200 µg/ml of rat gamma-globulin (Rockland) was performed prior to the addition of conjugated rat antibodies. Biotinylated antibodies were detected using streptavidin-PE-Cy5 purchased from BD Biosciences (554062) In some instances dead cells were excluded using the Fixable UV live/dead staining kit (Invitrogen) according to the manufacturer’s instructions

### Histology and Immunofluorescence

Spinal cord sectioning and haematoxylin/eosin (H+E) staining was performed essentially as previously described [7]. In some instances, luxol fast blue (LFB) staining was performed prior to H+E staining by fixing sections in ice-cold acetone for 10 minutes prior to incubating in a 0.1% LFB solution for 2 hours at 60°C. Slides were then rinsed in 70% ethanol then dH_2_O, and incubated in a 0.05% lithium carbonate solution in PBS for 5 seconds before rinsing in dH_2_O and then differentiation in 70% ethanol. Rinsing and differentiation was repeated until the grey matter of the spinal cord appeared colourless. Slides were then treated for H+E staining. Immunofluorescence was conducted essentially as previously described [32]. Briefly, sections were fixed in 100% cold acetone for 10 minutes and then air-dried and stored overnight at −20°C. All incubations were performed in a humid chamber at room temperature and all washes were involved three changes through 1X TBS for 2 minutes per wash. Slides were rehydrated in 1X TBS, blocked by incubating with 2% normal mouse serum and 2% normal goat serum in TBS for 30 minutes then washed. A diluted anti-mouse CD4 monoclonal antibody (BD Biosciences) or isotype-matched control antibody (BD Biosciences) was added to appropriate sections and these incubated for 1 hour. Slides were washed 3X followed by addition of anti-rat Alexa Fluor-647 (Molecular Probes) to the sections, which were then incubated for 45 minutes at room temperature followed by 3 washes. Slides were then air-dried and coverslips mounted using fluorescence-preserving medium (Vectashield, Vector Laboratories).

### In vitro T Cell Activation and Proliferation Assays

T cells harvested from the spleens of 6 week old mice were activated by culturing on trays pre-coated with anti-CD3 (clone # 2C11) at 10 µg/ml in the presence of 1 µg/ml anti-CD28 at 1×10^6^ cells/ml in 200 µl complete RPMI (RPMI 1640 supplemented with 10% FCS, 54 pM beta mercaptoethanol, 0.4 µM L-Glutamine and penicillin/gentamicin). In experiments where cell division was measured, cells were labeled with CFSE prior to culture as previously described [32]. Cells were activated for 3 days at 37°C in 5% CO_2_. Cells were then harvested and stained for flow cytometric analysis as described above.

### In vivo T Cell Proliferation Assay

BrdU (Sigma, Castle Hill, Australia) was added to drinking water (0.8 mg/ml) of mice on day 6 post-immunization until day 9 post-immunization. Proliferation was analyzed *ex vivo* by paraformaldehyde mediated fixation and permeabilization followed by FITC-labelled anti-BrdU (Becton Dickinson Immunocytometry, San Jose, CA) and flow cytometry as previously described [7].

### Detection of Apoptotic Cells by Flow Cytometry

This was performed essesntially as previously described [7], [61]. In brief, 2.5×10^5^ cells were suspended in 100 µl of incubation buffer (10 mM Hepes/NaOH pH 7.4, 140 nM NaCl, 5 mM CaCl_2_) with Annexin-V-Fluos (Roche, 20 µl per 1 ml of incubation buffer) and Propidium Iodide (Sigma Aldrich, 10 µl per 1 ml of incubation buffer) for 15 minutes before being centrifuged and re-suspended in incubation buffer for analysis. In some cases they were first stained with anti-CD4:PE-Cy7 antibodies as described above. Cells were analyzed within 30 minutes of labeling.

### In vivo Administration of PI3Kγ Inhibitor

Mice were treated with 30 mg/kg AS605240 in 100 µl of 0.5% methylcellulose/0.1% Tween20 in PBS by oral gavage every 12 hours as has been previously described [18].

### Statistical Analysis

The two-tailed unpaired Student’s T test was used for all statistical analyses with the following two exceptions: for EAE clinical disease analyses, two-way ANOVA analysis was performed; for analysis of the percentage of mice that recovered from EAE (Table 2), a chi squared test was employed. Statistical tests were performed using Graphpad Prism 5 software (Graphpad). *P* values of <0.05 were considered significant. In all cases * denotes *P*<0.05, ** denotes *P*<0.01 and *** denotes *P*<0.001.
